# Whole Pelvic Radiotherapy With Stereotactic Body Radiotherapy Boost vs. Conventionally Fractionated Radiotherapy for Patients With High or Very High-Risk Prostate Cancer

**DOI:** 10.3389/fonc.2020.00814

**Published:** 2020-05-29

**Authors:** Shih-Chang Wang, Wei-Chen Ting, Yun-Ching Chang, Ching-Chieh Yang, Li-Ching Lin, Hsiu-Wen Ho, Shou-Sheng Chu, Yu-Wei Lin

**Affiliations:** ^1^Department of Radiation Oncology, Chi Mei Medical Center, Tainan, Taiwan; ^2^Department of Radiation Oncology, Antai Medical Care Corporation Antai Tian-Sheng Memorial Hospital, Pingtung, Taiwan; ^3^Department of Nursing, Shu-Zen College of Medicine and Management, Kaohsiung, Taiwan; ^4^Department of Pharmacy, Chia-Nan University of Pharmacy and Science, Tainan, Taiwan; ^5^Institute of Biomedical Sciences, National Sun Yat-Sen University, Kaohsiung, Taiwan

**Keywords:** prostate cancer, SBRT, conventionally fractionated, high risk, radiotherapy

## Abstract

**Background:** Whole pelvic radiotherapy (WPRT) with stereotactic body radiotherapy (SBRT) boost has been shown to be effective in patients with high-risk prostate cancer (PC). However, no study has directly compared the efficacy of WPRT with SBRT boost with that of conventionally fractionated radiotherapy (CFRT). We compared the clinical outcomes between CFRT and WPRT with SBRT boost in patients with high or very high-risk PC (National Comprehensive Cancer Network definition).

**Methods:** In total, 132 patients treated with CFRT and 121 patients treated with WPRT followed by SBRT boost were retrospectively analyzed. For the CFRT group, the prescribed dose range was 74–79.2 Gray (Gy) administered at 1.8–2 Gy per fraction. For WPRT with SBRT boost, the prescribed doses were 45 Gy administered in 25 fractions to the whole pelvis followed by 21 Gy boost (3 fractions of 7 Gy each) to prostate and seminal vesicles. The overall survival (OS) and biochemical failure (Phoenix definition) free survival (bFFS) were assessed by using the Kaplan–Meier method or the Cox proportional hazards regression model. The gastrointestinal (GI) and genitourinary (GU) tract toxicity were assessed using the National Cancer Institute Common Terminology Criteria for Adverse Events (CTCAE) v3.0.

**Results:** The estimated 4-years overall survival in the CFRT and WPRT with SBRT boost groups was 91.6 and 97.7%, respectively (*P* = 0.18). The estimated 4-years biochemical failure-free survival in the CFRT and WPRT with SBRT boost groups was 89.1 and 93.9%, respectively (*P* = 0.41). No acute grade 3 or higher GI and GU toxicity was observed in both groups. Late grade 3 GI and GU toxicity occurred in 2.3 and 2.3% in the CFRT group, and in 1.7 and 0.8% in the WPRT with SBRT boost group, respectively. There was no significant between-group difference with respect to acute or late toxicity.

**Conclusions:** In patients with high or very high-risk localized PC, compared with CFRT, WPRT with SBRT boost resulted in similar biochemical-free and overall survival rate with minimal toxicity. WPRT with SBRT boost is a feasible option for patients with high or very high-risk PC.

## Introduction

Prostate cancer (PC) is the second most common malignancy in men. Globally, an estimated 1.1 million new cases of PC were diagnosed in 2012 (1). Risk stratification of patients with prostate cancer is critical to determine prognosis and guide treatment decision-making (2). High-risk disease accounts for ~15% of new cases of PC diagnosed in the US and up to 30% of those in Asia (3, 4). Patients with high or very high-risk PC are at a greater risk of treatment failure and impaired quality of life. Radiotherapy (RT) plays an important role in the management of patients with PC (5). Recent decades have witnessed substantial progress in radiation therapy techniques for PC. Several randomized controlled trials have shown that dose escalation may help achieve better biochemical control (6–9). However, dose escalation and use of three-dimensional conformal radiation therapy (3D-CRT) is associated with a higher incidence of toxicity. Currently, intensity modulated radiation therapy (IMRT) has increasingly replaced 3D-CRT in order to reduce dose exposure to anatomically contiguous normal organs and to minimize treatment-related toxicity (10, 11).

According to the low alpha/beta ratio of PC cells, increased fraction size RT may help improve therapeutic control without a significant increase in late toxicity to adjoining normal tissues (12, 13). Stereotactic body radiotherapy (SBRT), a new technique to deliver highly conformal and high-dose radiation, is also being increasingly used for treatment of PC (14–16). SBRT not only offers radiobiological advantage but also provides the convenience of non-invasiveness and shorter treatment duration. A recent series of studies have investigated incorporation of SBRT boost in conjunction with whole pelvis radiotherapy (WPRT) for treatment of high-risk PC (17–21). The preliminary results demonstrated promising biochemical control rate with minimal toxicity. However, no study has directly compared the efficacy of conventionally fractionated radiotherapy (CFRT) with that of WPRT with SBRT boost.

The main aims of this study were to compare the efficacy and toxicity between CFRT and WPRT with SBRT boost in patients with high or very high-risk localized PC.

## Methods and Materials

### Patient Characteristics

Patients who were newly diagnosed with high or very high-risk PC [National Comprehensive Cancer Network (NCCN) definition] and were treated with CFRT or WPRT with SBRT boost at the Chi Mei Medical Center between July 2009 and May 2018 were enrolled in this retrospective study. All patients had biopsy confirmed adenocarcinoma of the prostate. The exclusion criteria were as follows: (1) stage IV disease such as T4, nodal involvement, or distant metastasis; (2) prior pelvic RT or radical prostatectomy; (3) missing data. This study was approved by our institutional review board.

### Treatment

All patients underwent computed tomography simulation; fusion magnetic resonance images were utilized for accurate contour delineation. For patients treated with CFRT, IMRT was delivered using 6- or 10-MV photons. The prescription doses were 45 Gray (Gy) for WPRT and 74–79.2 Gy for prostate and seminal vesicles at the rate of 1.8–2.0 Gray per fraction. For patients who received WPRT followed by SBRT boost, transrectal implantation of four fiducials was performed in the prostate prior to radiotherapy. The prescription dose of WPRT was 45 Gy administered in 25 fractions. The dose of SBRT boost was 21 Gy (3 fractions of 7 Gy) administered to prostate and seminal vesicles by CyberKnife (Accuracy) system on every other day. The biologically effective dose (BED) were 151–165 Gy for CFRT and 192 Gy for WPRT with SBRT boost when assuming an alpha/beta ratio of 1.8 for prostate cancer (13). The majority of the patients received long-term androgen deprivation therapy (ADT), which was prescribed at the discretion of the physician. Treatment with luteinizing hormone-releasing hormone (LH-RH) agonists, anti-androgen agents, or a combination of these agents was also allowed.

### Treatment Plan Criteria for WPRT With SBRT Boost

For WPRT, the prostate gland, entire seminal vesicles, and the area of extracapsular extension were defined as the clinical target volume (CTV) 1. CTV2 includes the external iliac, internal iliac, presacral, and obturator nodes. The planning target volume (PTV) 1 was created by adding a 7 mm margin to the CTV1 throughout except posteriorly by the rectum where a 5 mm margin was used. The PTV2 was extended 7 mm in all directions. The constraint was <17% of the rectal volume to receive more than 42 Gy (V42 < 17%) for rectum, and <40% of the urinary bladder volume to receive more than 40 Gy (V40 < 40%) for bladder. Bowel constraints were <0–1 cm^3^ of the bowel volume to receive more than 52–54 Gy (V54–52 < 0–1 cm^3^) and mean bowel dose <23.5 Gy (mean dose < 23.5 Gy). A minimum of 95% of the prescription dose was assured to cover 100% of the PTV. The WPRT treatment plans were generated on Varian Eclipse treatment planning system (version 8.6.10, Varian Medical Systems, Palo Alto, CA, USA). For SBRT boost, the prostate gland, entire seminal vesicles, and area of extracapsular extension were defined as CTV. The PTV was extended 5 mm beyond the CTV in all directions, except in the posterior direction, wherein it was extended 3 mm. The rectum constraints were <1 cm^3^ of the rectum volume to receive more than 20 Gy (V20 < 1 cm^3^) and <17% of the rectal volume to receive more than 14.5 Gy (V14.5 < 17%). The bladder constraints were <5 cm^3^ of the bladder volume to receive more than 21 Gy (V21 < 5 cm^3^) and <25% of the urinary bladder volume to receive more than 14.5 Gy (V14.5 < 25%). The penile bulb constraint was <50% of the penile bulb volume to receive more than 16.5 Gy (V16.5 < 50%). A minimum of 95% of the prescription dose was assured to cover 95% of the PTV after prescription to the 80% (or higher) isodose line. The SBRT boost treatment plans were generated on MultiPlan (version 2.2.0, Accuracy Incorporated, Sunnyvale, CA, USA) (18).

### Follow-Up

All patients were examined 4 weeks after the final treatment, every 3 months for the first 2 years, and every 6 months thereafter. At each evaluation, the prostate-specific antigen (PSA) level was obtained, and the toxicity was assessed using the National Cancer Institute Common Terminology Criteria for Adverse Events v3.0. Biochemical failure was defined according to the Phoenix definition (increase in PSA level by at least 2 ng/mL from nadir) (22). Biochemical failure-free survival (bFFS) and overall survival (OS) were evaluated from the 1st day of radiation therapy to the date of event. Time to PSA nadir was evaluated from completion of treatment to the date of event.

### Statistical Analysis

The Chi-Squared test was used to compare the distribution of categorical variables, and ANOVA was used to compare continuous variables. The Kaplan–Meier method was used to construct the survival curves. The survival curves were compared using the log-rank test. For univariate and multivariate analyses, the Cox proportional hazards regression model was applied to assess the factors to estimate survival outcomes. *P* < 0.05 were considered indicative of statistical significance. All statistical analyzes were performed using SPSS version 19.0 (SPSS Inc., Chicago, IL).

## Results

### Patient Characteristics

**Figure 1:**
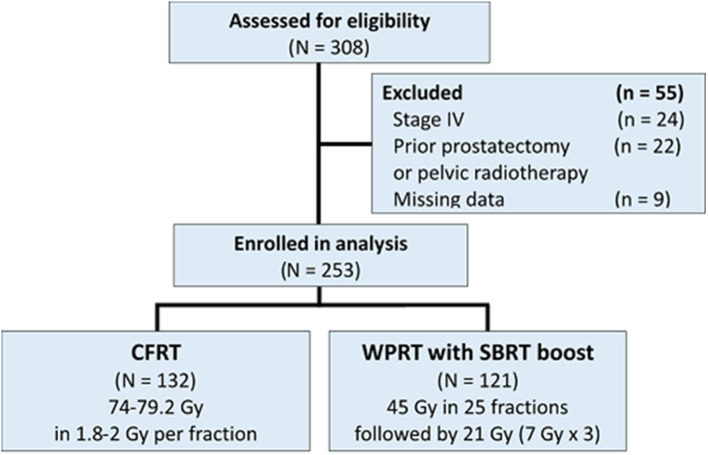
Schematic illustration of the study design and patient-selection criteria.

A total of 308 patients were initially found eligible for this study. Of these, 55 patients were excluded [stage IV disease (*n* = 24); prior pelvic RT or radical prostatectomy (*n* = 22); missing data (*n* = 9)]. Finally, a total of 253 patients were included in the analysis (Figure 1); of these, 132 patients were treated with CFRT while 121 patients were treated with WPRT followed by SBRT boost. The patient characteristics are summarized in Table 1. There were no significant between-group differences with respect to age, performance status (Eastern Cooperative Oncology Group, ECOG), T stage, PSA level, Gleason score, or NCCN risk group. Use of ADT was significantly more frequent in the CFRT group (97.7% in CFRT; 91.7% in WPRT with SBRT boost; *P* = 0.044). The mean duration of ADT was longer in the CFRT group than WPRT with SBRT boost group (CFRT vs. WPRT with SBRT boost: 30.6 months vs. 24.6 months, *P* = 0.001). More than half of all patients had higher T stage (T3), increased PSA level (>20 ng/mL), and Gleason score (≥8) in both groups.

**Table 1:** Patients characteristics.

	**Numbers of patients (%)**		
**Characteristics**	**CFRT** **(*n* = 132)**	**WPRT with SBRT boost** **(*n* = 121)**	***P*-value**
**Age (years)**			0.066
≥70	103 (78.0)	82 (67.8)	
<70	29 (22.0)	39 (32.2)	
Median, range	75 (56−89)	73 (50−90)	
**ECOG PS**			0.393
0–1	127 (96.2)	112 (92.5)	
2	4 (3.0)	6 (5.0)	
3	1 (0.8)	3 (2.5)	
**T stage**			0.600
T1	3 (2.3)	1 (0.8)	
T2a	10 (7.6)	13 (10.7)	
T2b–T2c	29 (22.0)	27 (22.3)	
T3a	48 (36.4)	36 (29.8)	
T3b	42 (31.8)	44 (36.4)	
**PSA level (ng/ml)**			0.253
<10	19 (14.4)	27 (22.3)	
10–20	28 (21.2)	25 (20.7)	
>20	85 (64.4)	69 (57.0)	
**Gleason score**			0.570
≤ 6	27 (20.5)	22 (18.2)	
7	35 (26.5)	38 (31.4)	
8	28 (21.2)	23 (19.0)	
9	38 (28.8)	30 (24.8)	
10	4 (3.0)	8 (6.6)	
**NCCN risk group**			0.929
High	76 (57.6)	69 (57.0)	
Very high	56 (42.4)	52 (43.0)	
**ADT**			0.044
Yes	129 (97.7)	111 (91.7)	
No	3 (2.3)	10 (8.3)	
**Duration of ADT (months)**			0.001
<18	36 (27.2)	34 (28.1)	
18–36	55 (41.7)	71 (58.7)	
>36	41 (31.1)	16 (13.2)	
Mean, range	30.6 (5.7–83.0)	24.6 (0.9–54.6)	

*CFRT, conventionally fractionated radiotherapy; WPRT, whole pelvis radiotherapy; SBRT, stereotactic body radiotherapy; ECOG PS, Eastern Cooperative Oncology Group Performance Status; ADT, androgen deprivation therapy*.

### Outcomes for Biochemical Failure-Free and Overall Survival

**Figure 2:**
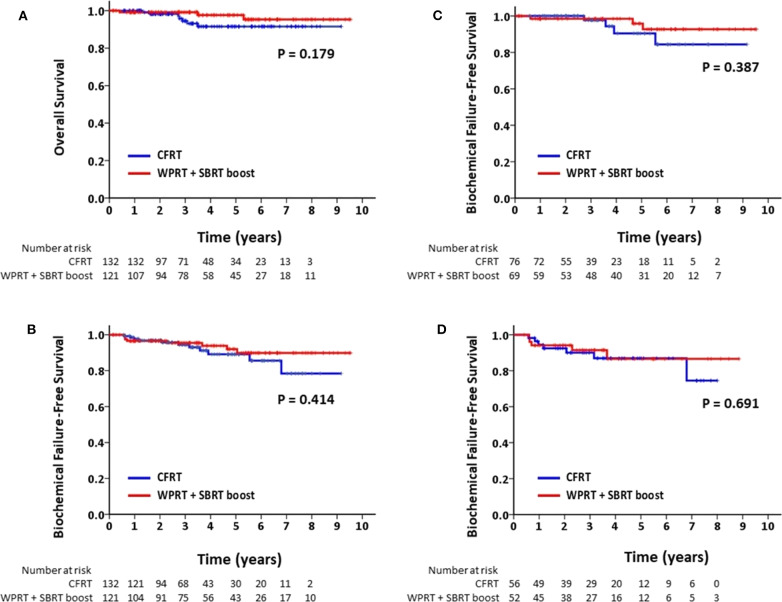
Kaplan–Meier analysis of **(A)** overall survival for all patients; **(B)** biochemical failure-free survival for all patients; **(C)** biochemical failure-free survival for patients with high-risk PC; and **(D)** biochemical failure-free survival for patients with very high-risk PC.

The mean follow-up time for patients in the CFRT group and the WPRT with SBRT boost group was 41.4 and 48.5 months, respectively. Figure 2 shows the Kaplan–Meier curves for survival. The estimated 4-years OS in the CFRT group and WPRT with SBRT boost group was 91.6 and 97.7%, respectively (*P* = 0.18). The estimated 4-years bFFS was 89.1 and 93.9%, respectively (*P* = 0.41). On sub-group analysis disaggregated by risk status, there was no significant difference between the 4-years bFFS of high-risk (90.4 vs. 98.5%; *P* = 0.39) or very high-risk patients with PC (86.9 vs. 86.6%; *P* = 0.69) in the CFRT and WPRT with SBRT boost groups. Table 2 presented the results of the univariate and multivariate analyses. The multivariate analysis revealed that patients with very high risk group disease (HR, 3.45; 95% CI: 1.25–9.47; *p* = 0.016) or without use of ADT (HR, 9.57; 95% CI: 2.49–36.88; *p* = 0.001) was associated with poor bFFS. The treatment modality either CFRT or WPRT with SBRT boost was not a prognostic factor for the bFFS (HR, 0.64; 95% CI: 0.25–1.63; *p* = 0.351).

**Table 2:** Cox regrssion analysis of biochemical failure-free survival (bFFS).

**Characteristics**	**Univariate**			**Multivariate**		
	**HR**	**(95% CI)**	***p*-value**	**HR**	**(95% CI)**	***p*-value**
**Age**						
<70	1.00					
≥70	1.25	(0.45–3.49)	0.666			
**ECOG PS**						
0–1	1.00					
≥2	3.51	(0.80–15.47)	0.097			
**T stage**						
T1–T2a	1.00					
T2b–T2c	1.08	(0.28–4.20)	0.907			
T3	0.52	(0.14–1.92)	0.325			
**PSA level (ng/ml)**						
<10	1.00					
10–20	0.59	(0.10–3.56)	0.568			
>20	1.24	(0.36–4.31)	0.737			
**Gleason score**						
≤ 6	1.00					
7	3.42	(0.40–29.36)	0.262			
≥8	5.92	(0.77–45.30)	0.087			
**NCCN risk group**						
High	1.00			1.00		
Very High	2.64	(1.03–6.73)	0.042	3.45	(1.25–9.47)	0.016
**ADT**						
Yes	1.00			1.00		
No	5.00	(1.45–17.32)	0.011	9.57	(2.49–36.88)	0.001
**Duration of ADT (months)**						
<18	1.00					
18–36	0.45	(0.15–1.33)	0.148			
>36	0.68	(0.22–2.14)	0.512			
**Treatment modality**						
CFRT	1.00			1.00		
WPRT with SBRT boost	0.68	(0.27–1.71)	0.684	0.64	(0.25–1.63)	0.351

### PSA Response

**Figure 3:**
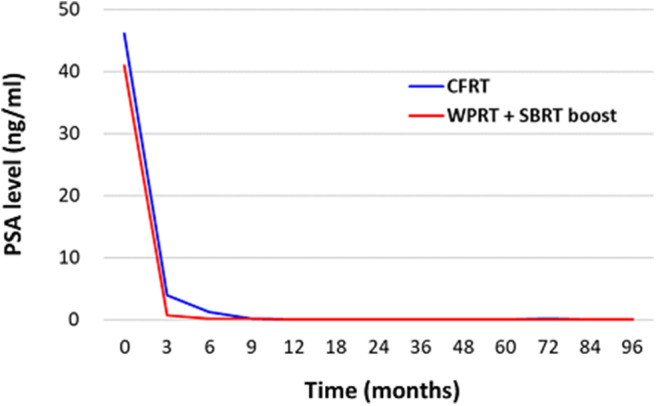
PSA response after treatment.

Figure 3 illustrates the PSA response following treatment. The mean PSA level before treatment was 46.16 ng/mL in the CFRT group and 40.93 ng/mL in the WPRT with SBRT boost group. Post-treatment, the mean PSA levels (excluding biochemical failures) at 3, 6, 9, 12, 18, 24, 36, 60, and 84 months were 3.94, 1.28, 0.16, 0.13, 0.10, 0.10, 0.11, 0.09, and 0.09 ng/mL in the CFRT group, and 0.73, 0.24, 0.14, 0.11, 0.07, 0.05, 0.06, 0.06, and 0.10 ng/mL in the WPRT with SBRT boost group. The mean time to nadir of PSA was 9.7 months in the CFRT group, and 10.7 months in the WPRT with SBRT boost group (*P* = 0.33).

### Toxicities

No treatment-related deaths were observed in this study. The observed acute and late GI or genitourinary (GU) adverse events are provided in Table 3. There was no significant between-group difference with respect to acute or late toxicity. None of the patients in the CFRT group developed acute grade 3 GI toxicity; however, one event of acute grade 3 GU toxicity (0.8%) was observed. None of the patients in the WPRT with SBRT boost group developed acute grade 3 GI or GU toxicity. Late grade 3 GI and GU toxicity occurred in 3 (2.3%) and 3 (2.3%) patients in the CFRT group, and in 2 (1.7%) and 1 (0.8%) patients in the WPRT with SBRT boost group, respectively.

**Table 3:** GI and GU toxicity.

	**CFRT** **(*n* = 132)**	**WPRT with SBRT boost** **(*n* = 121)**	
**Grade**	**Acute GI toxicity (%)**		***P*****-value**
0	49 (37.1)	41 (33.9)	0.308
1	68 (51.5)	58 (47.9)	
2	15 (11.4)	22 (18.2)	
3	0 (0)	0 (0)	
4	0 (0)	0 (0)	
	**Acute GU toxicity (%)**		
0	29 (22.0)	34 (28.1)	0.218
1	64 (48.5)	45 (37.2)	
2	38 (28.8)	42 (34.7)	
3	1 (0.8)	0 (0)	
4	0 (0)	0 (0)	
	**Late GI toxicity (%)**		
0	108 (81.8)	94 (77.7)	0.223
1	15 (11.4)	23 (19.0)	
2	6 (4.5)	2 (1.7)	
3	3 (2.3)	2 (1.7)	
4	0 (0)	0 (0)	
	**Late GU toxicity (%)**		
0	69 (52.3)	67 (55.4)	0.527
1	39 (29.5)	29 (24.0)	
2	21 (15.9)	24 (19.8)	
3	3 (2.3)	1 (0.8)	
4	0 (0)	0 (0)	

*GI, gastrointestinal; GU, genitourinary*.

## Discussion

To the best of our knowledge, this is the first study that directly compared the outcomes of CFRT with those of WPRT with SBRT boost in patients with high-risk PC. The results demonstrated no significant between-group difference with respect to OS, biochemical control, or toxicity.

Some phase III RCTs had supported the use of moderately hypofractionated RT, which is another modality that provides higher dose per fraction with a shorter course. However, not all of these trials included patients with high-risk PC. In a study by Hoffman et al. 72 Gy administered in 2.4 Gy fractions over 6 weeks achieved similar disease control as that achieved with 75.6 Gy administered in 1.8-Gy fractions over 8.4 weeks; however, patients in the high-risk group accounted for only 1% of the study population (23). The CHHiP trial, the largest RCT, recruited 385 (12%) high-risk PC patients and demonstrated that 60 Gy administered in 20 fractions over 4 weeks is non-inferior to 74 Gy administered in 37 fractions over 7.4 weeks (24). A study by Pollack et al. enrolled 34% patients with high-risk PC; the results showed no significant difference between hypofractionation RT (70.2 Gy in 26 fractions) and CFRT (76 Gy in 38 fractions) (25). The HYPRO trial randomized 820 men with intermediate-risk (26%) and high-risk (74%) disease to hypofractionated group (64.6 Gy in 19 fractions) or conventional fractionated group (78 Gy in 39 fractions); similar 5-years relapse-free survival rate was reported in both groups (80.5 and 77.1%, respectively) (26).

Arcangeli et al. reported a study wherein 168 patients with high-risk disease were randomized to 62 Gy at 3.1 Gy per fraction over 5 weeks or 80.0 Gy in 40 fractions over 8 weeks (27). The 5-years biochemical control rate was 85% in the hypofractionated group and 79% in the conventional fractionated group (*P* = 0.065). For patients with high-risk disease, our study showed 5-years biochemical failure-free survival of ~90%, which is somewhat higher than the results reported by Arcangeli et al. This may be attributable to two reasons. First, in the study by Arcangeli et al. patients received 9 months of ADT, which is much shorter than current suggestion of long-term (2–3 years) hormone therapy for high-risk disease. In our study, the mean duration of ADT was >2 years. Second, the study by Arcangeli et al. adopted 3D-CRT treatment planning, rather than IMRT. IMRT is known to be more effective than 3D-CRT with respect to target coverage and dose distribution. Therefore, the dose delivered to target volume in 3D-CRT planning may have been compromised.

HYPO-RT-PC trial, recently published in June 2019, is the first RCT comparing the extremely hypofractionation to conventional fractionation in men with intermediate-to-high risk prostate cancer (28). It demonstrated extremely hypofractionation (42.7 Gy in 7 fractions) is non-inferior to conventional fractionation radiotherapy (78.0 Gy in 39 fractions) regarding 5-years failure-free survival outcome. However, the majority of the HYPO-RT-PC population was intermediate risk, and only 11% high-risk. Besides, T3b or PSA >20 ng/mL was not allowed to recruit in this study. It may not reflect to the actual high-risk population. Therefore, it still need more prospective evidence supporting use of extremely fractionated RT in high-risk localized prostate cancer.

There are few studies for SBRT in high-risk prostate cancer with whole pelvic radiotherapy (WPRT), or so-called elective nodal irradiation (ENI). Traditionally, patients with high-risk prostate cancer receive WPRT, and such regimens have applied to large-scale clinical trials such as RTOG 8531, RTOG 9202, RTOG 9413, and EORTC 22863 (29–32). Though conventionally fractionated ENI has been delivered safely on several clinical trials, little data exist concerning the use of ENI as part of a hypofractionated treatment regimen. The FASTR trial included high-risk patients treated with 40 Gy in five fractions to the prostate and 25 Gy in five fractions to ENI. The trial was terminated early because of the higher late grade 3 GI or GU toxicity (33). However, in SATURN trial, using ENI with SBRT (40 Gy in 5 weekly fractions to the prostate and 25 Gy in five fractions to pelvic lymph nodes and seminal vesicles) is feasible and may lead to an improvement in biochemical control and the PSA response, without an increase in late GI or GU toxicity (34). Tata Memorial Center also reported SBRT with 35–37.5 Gy to prostate and 25 Gy to pelvic nodal regions in 5 fractions for high-risk, very high-risk and node-positive PC. Also, there was no increase in acute or late gastrointestinal toxicity with prophylactic pelvic nodal radiotherapy (35).

Extreme or moderately hypofractionated RT, which delivers higher dose for each fraction, may address the concerns pertaining to potentially worse late toxicity resulting from high-dose treatment. Datta et al. conducted a systematic review and meta-analysis of 10 trials (8,146 patients) that compared conventional vs. hypofractionated RT (36). The incidence of acute GU toxicity, late GU toxicity, and GI toxicity was not significantly different between the two modalities. Only the incidence of acute ≥ grade 2 GI toxicity was 9.1% less with conventionally fractionated RT (*P* < 0.001). In our study, the incidence of ≥ grade 2 acute GI toxicity in the WPRT with SBRT boost group (18.2%) was slightly higher than that in the CFRT group (11.4%); however, the between-group difference was not statistically significant. In the WPRT with SBRT boost group, the incidence of late grade 3 GI or GU toxicity was <2%. Multiple factors, such as use of IMRT, synchronous tracking system of CyberKnife, and the low alpha/beta ratio of PC may have contributed to the satisfactory outcomes of WPRT with SBRT boost.

The technique of SBRT boost offers two important advantages over CFRT. First, in our study, the daily RT treatment was reduced from 37 to 44 fractions in CFRT to 28 fractions in WPRT with SBRT boost. It represents a decrease of ~30% or 3 weeks of treatment duration. The shorter treatment course is more convenient to many patients who reside far away from the radiotherapy facility. Second, many studies have demonstrated that the treatment costs of SBRT are lower than those of CFRT (37–39). This would be cost effective and encourage patients to receive treatment.

In this study, WPRT with SBRT boost in patients with high-risk or very high-risk prostate cancer yielded biochemical control rates and toxicity profiles that are comparable to those with CFRT modality. We acknowledge the limitations of this retrospective trial. The risk of selection bias cannot be ruled out in the retrospective study. The number of patients and the duration of follow-up were still limited. Besides, the status of comorbidities and toxicity profiles would be more precisely by Charlson comorbidity index and patient self-reported outcomes.

## Conclusion

Here, we reported the largest study that assessed the combined use of WPRT with SBRT boost for patients with high or very high-risk PC and directly compared the clinical outcomes with those of CFRT. Compared with CFRT, WPRT with SBRT boost resulted in similar clinical outcomes with comparable OS, PSA control rate, and minimal toxicity. It is a feasible and shorter treatment option for patients with high or very high-risk PC. The conclusion is meant as hypothesis-generating only. A long-term follow-up would provide a better assessment of biochemical control and toxicity profile. Prospective randomized trials are encouraged to establish the role of WPRT with SBRT boost and confirm its equivalence to other fractionation schemes in high-risk and very high-risk prostate cancer.

## Data Availability Statement

The datasets generated for this study are available on request to the corresponding author.

## Ethics Statement

The studies involving human participants were reviewed and approved by Chi Mei Medical Center. The patients/participants provided their written informed consent to participate in this study.

## Author Contributions

S-CW conceived and designed the study, collected, analyzed, and interpreted the data, prepared the draft and gave final approval of the version to be submitted. W-CT, Y-CC, C-CY, L-CL, H-WH, and S-SC interpreted and undertook data analysis and carried out clinical revision of the data. Y-WL conceived the study, analyzed and interpreted the data, gave final approval of the version to be submitted. All authors read and approved the final manuscript.

## Conflict of Interest

The authors declare that the research was conducted in the absence of any commercial or financial relationships that could be construed as a potential conflict of interest.
